# Arbuscular mycorrhiza-induced growth promotion and disease resistance are fine-tuned by growth-defense tradeoffs in *Lotus japonicus* and tomato

**DOI:** 10.5511/plantbiotechnology.25.0220a

**Published:** 2025-09-25

**Authors:** Yuka Higashi, Hinako Ambiru, Hikaru Saito, Mayumi Egusa, Chihiro Miura, Takaya Tominaga, Hironori Kaminaka

**Affiliations:** 1 Department of Agricultural Science, Graduate School of Sustainable Science, Tottori University, Tottori, Tottori 680-8553, Japan; 2 Faculty of Agriculture, Tottori University, Tottori, Tottori 680-8553, Japan; 3 The United Graduate School of Agricultural Science, Tottori University, Tottori, Tottori 680-8553, Japan; 4 Division of Biological Science, Graduate School of Science and Technology, Nara Institute of Science and Technology, Ikoma, Nara 630-0192, Japan

**Keywords:** arbuscular mycorrhizal symbiosis, growth-defense tradeoffs, *Lotus japonicus*, mycorrhiza-induced resistance, tomato

## Abstract

Arbuscular mycorrhizal fungi (AMF) are representative symbiotic partners of plants, and trade nutrients with them. This symbiotic association confers plants with the agronomically beneficial traits such as plant growth promotion and stress tolerance. Arbuscular mycorrhizae (AM) are divided into two morphotypes, the *Arum*-type and the *Paris*-type, based on fungal structures within the host plant cells. Although the phylogeny of host plants typically determines the AM morphotype, the AMF, *Rhizophagus irregularis* and *Gigaspora margarita*, can form *Arum*-type AM and *Paris*-type AM, respectively, in tomato (*Solanum lycopersicum*). In this study, the traits resulting from the AM symbiosis and root transcriptomes between *Lotus japonicus* and tomato inoculated with these two phylogenetically distal AMF were compared. In *L. japonicus*, *Arum*-type AMs formed when colonized by both AMF, as expected. Shoot growth in both plants was significantly promoted when inoculated by these AMF, although the impact of *G. margarita* was greater than that by *R. irregularis* colonization. A transcriptome analysis of both plants inoculated by the two AMF strongly suggested changes in the expression levels of genes associated with defense response. AMF inoculation induced resistance against *Fusarium* diseases in both plants, but the level of disease resistance in *Rhizophagus*-colonized plants was higher than in *Gigaspora*-colonized plants. Thus, the colonized AMF identity, and not the AM morphotype, determines the level of AM-induced traits, plant growth promotion and disease resistance. Negative relationships between these two traits would exist as a growth-defense tradeoff to fine-tune the balance in response to limited resources, and to optimize fitness.

## Introduction

Plants are associated with diverse microbes, regardless of the positive or negative association, which ultimately impacts plant fitness. The association with symbionts in a plant’s microbiota offers it an advantageous adaptation to various environmental conditions, or to changes in them. Over 80% of terrestrial plants form symbiotic associations with mycorrhizal fungi in their roots (Martin and van der Heijden 2024). Among these, arbuscular mycorrhizal fungi (AMF), which are the members of the Glomeromycota, are the most prevalent symbiotic partner, being found in over 70% of terrestrial plants (Brundrett and Tedersoo 2018). During symbiosis, a reciprocal exchange of nutrients occurs between host plants and AMF, in which plants supply fixed carbon that AMF can utilize, and, in return, AMF allocate minerals in soil, particularly phosphates (Smith and Smith 2011). Therefore, AMF colonization in plant roots is able to promote growth, even when phosphate is scarce. To establish this symbiotic association, plants supply as much as 20% of all carbon assimilated during photosynthesis (Jakobsen 1995). In addition, AMF enhance the host plant’s resistance to abiotic and biotic stresses (Shi et al. 2023). AMF root colonization can increase resistance against both belowground and aboveground pathogens, termed phenomenon referred to as mycorrhiza-induced resistance (MIR) (Fiorilli et al. 2024).

Mycorrhizae are classified based on their structure and function (Martin and van der Heijden 2024). Arbuscular mycorrhizae (AM) are further divided into two morphotypes, the *Arum*-type and the *Paris*-type, based on their fungal structures (Dickson 2004). *Arum*-type AM with intercellular hyphae in the root cortex and arbuscules in cortical cells are typically found in cultivated crops, while trees and herbaceous plants typically produce *Paris*-type AM, penetrating cells with intracellular hyphae and forming arbuscules or hyphal coils in cortical cells (Dickson 2004). The phylogeny of the host plant basically determines the AM morphotype, although some exceptions exist (Dickson et al. 2007; Hong et al. 2012; Smith and Smith 1997; Tominaga et al. 2022). In tomato (*Solanum lycopersicum*), *Rhizophagus irregularis* and *Gigaspora margarita*, both AMF, can form an *Arum*-type AM and a *Paris*-type AM, respectively (Cavagnaro et al. 2001). Using these tomato-AMF combinations, we previously compared the root transcriptomes of *Arum*-type and *Paris*-type AMs (Tominaga et al. 2022). *Paris*-type AM-specific transcriptional responses involved in defense and gibberellin biosynthesis were observed, suggesting that different transcriptional responses in the two AM types contributed to the fine-tuning of mutualism between tomato roots and these AM fungi to optimize the host plant growth (Tominaga et al. 2022). However, the possibility that the different transcriptional responses that were observed between *Arum*-type and *Paris*-type AMs were simply caused by responses to phylogenetically distal AMF, and not by differences in AM morphotypes, cannot be excluded.

In this study, comparative analyses were conducted between *Lotus japonicus*, a leguminous model plant, and tomato, a model horticultural plant, inoculated with two AMF, *R. irregularis* and *G. margarita*. As previously noted, these AMF form different types of AMs in tomato, although only *Arum*-type AM form in *L. japonicus* (Tominaga et al. 2021; Venice et al. 2021). The results revealed that AM morphotype does not seem to be involved in plant transcriptional responses and traits commonly induced by these two phylogenetically distal AMF. In addition, negative relationships between plant growth promotion and induced disease resistance caused by AMF root colonization were found in both plants.

## Materials and methods

### Plant and fungal materials

Seeds of *L. japonicus* (accession Miyakojima MG-20) were obtained from the National BioResource Project Lotus/Glycine (Legume base; Miyazaki, Japan). Seeds of tomato cv. Micro-Tom were obtained from the National BioResource Project Tomato (TOMATOMA; Tsukuba, Japan). Tomato cv. Sugar Lump and cv. Momotaro seeds were purchased from Sakata Seed (Yokohama, Japan) and Takii Seed (Kyoto, Japan), respectively. Axenic spores of *R. irregularis* DAOM197198 (Grade-C) were purchased from Agronutrition (Carbonne, France). *Gigaspora margarita* K-1 (MAFF520052) was obtained from NARO Genebank (Tsukuba, Japan). *Gigaspora margarita* was propagated in red clover (*Trifolium pratense*), and the collection and surface sterilization of spores were conducted as described previously (Tominaga et al. 2023).

### Plant growth conditions and AMF inoculation

To induce germination, the seeds of *L. japonicus* and tomato were washed with 70% ethanol, rinsed twice with sterilized distilled water, then incubated in 1.5% (v/v) NaClO solution for 15 min. After the seeds were rinsed twice with sterilized distilled water, the surface-sterilized seeds were incubated on plastic Petri dishes with two sheets of filter paper immersed in sterilized distilled water, and placed under a 14-h photoperiod (80 µmol m^−2^ s^−1^) at 25°C for six days in a growth chamber (BiOTRON NK System; Nippon Medical and Chemical Instrument, Tokyo, Japan). Three *L. japonicus* or tomato seedlings were transplanted to inoculation pots filled with 300 ml of sterilized culture soil (1 : 2 mixture of Shibanome soil and river sand) containing 1/5 Hoagland’s solution (20-µM inorganic phosphate), prepared according to Tominaga et al. (2020). For the inoculation of AMF, *R. irregularis* axenic spores (2000 spores/plant) or *G. margarita* sterilized spores (20 spores/plant) were mixed with the culture soil prior to transplanting. Unless stated otherwise, seedlings were grown under the same controlled environmental conditions that were used for germination. Tomato was cultivated under a 14-h photoperiod at 25°C, but three light intensities were tested: high light intensity (330 µmol m^−2^ s^−1^), moderate light intensity (80 µmol m^−2^ s^−1^), or low light intensity (5 µmol m^−2^ s^−1^). After 5 weeks, roots were harvested and fixed in FAA solution (5% formaldehyde, 5% acetic acid, 45% ethanol). The fresh weight of shoots was measured on an electronic balance (ME204; Mettler-Toledo, OH, USA).

### Observation and quantification of root colonization by AMF

Root colonization by AMF was visualized by trypan blue staining, according to a previous study (Tominaga et al. 2020). Photos of stained root samples were taken under a light microscope (BX53; Olympus, Tokyo, Japan) equipped with a digital camera (DP27; Olympus). The rate of colonization by AMF (%) was calculated based on the frequencies of intraradical hyphae and arbuscules, as described previously (Tominaga et al. 2020).

### RNA-sequencing and transcriptome analysis

Total RNA from *L. japonicus* roots that were inoculated with *R. irregularis* or *G. margarita*, and then cultured for 6 weeks, was prepared using RNAiso Plus (TAKARA, Shiga, Japan) after pretreatment with Fruit-mate for RNA Purification (TAKARA), according to the manufacturer’s instructions. Roots from three seedlings were used for each repetition per treatment, including the control, which were plants that were not inoculated by AMF. DNAse I treatment, preparation of sequencing libraries, and sequencing with strand-specific and paired-end reads (150 bp) by DNBSEQ-G400RS were performed by Genome-Lead Co. (Takamatsu, Japan).

The filtering, trimming, mapping, and counting of raw reads were performed as previously described, but with several modifications (Tominaga et al. 2022). Filtering and trimming of the paired-end raw reads were performed using fastp v.0.22.0 (Chen et al. 2018). The filtered reads were aligned against the *L. japonicus* MG20 v3.0 genome assembly obtained from the Lotus Base (https://lotus.au.dk/) using STAR v2.7.10b (Dobin et al. 2013) with the paired-end option. Aligned reads were counted by feature Counts v.2.0.6, and differentially expressed genes (DEGs) were identified by edgeR v.4.2.2 (Robinson et al. 2010) using R v.4.5.2 with a cut-off of |log_2_ fold change (FC)| ≥1 and false discovery rate (FDR) <0.05. The results of sequencing and mapping are summarized in Supplementary Table S1. The dataset of DEGs obtained for tomato was drawn from our previous study (Tominaga et al. 2022). The Venn diagram was drawn using the online tool “molbiotools” (https://molbiotools.com/).

### Gene ontology analysis

Gene ontology (GO) terms were identified using EggNOG-mapper (Buchfink et al. 2015; Huerta-Cepas et al. 2017, 2019) v.2.1.3 against Lotusjaponicus_MG20_v3.0_proteins.fa for *L. japonicus* and ITAG4.0_proteins.fasta for *S. lycopersicum*. The GO enrichment analysis of DEGs was performed using the topGO v.2.56.0 (https://bioconductor.org/packages/release/bioc/html/topGO.html) in the R environment. Overrepresented GO terms were identified with a cut-off elim-Kolmogorov–Smirnov (KS) value of <0.01.

### *Fusarium* inoculation test

For *L. japonicus*, germination, transplanting, AMF inoculation, and cultivation followed the procedures outlined above, except for the number of transplanted seedlings and the number of inoculated AMF spores: six seedlings, and *R. irregularis* (1000 spores/plant) or *G. margarita* (20 spores/plant) were used. After cultivation for 6 weeks, each seedling was inoculated with 1 ml of a bud cell suspension (5×10^7^ cells/ml) of *Fusarium solani-melongenae* (FSO) MAFF240020, a causal agent of *Fusarium* root rot of *L. japonicus* (Takeuchi et al. 2007), by adding inoculum into soil. Disease severity was evaluated based on the shoot fresh weight 3 weeks after *Fusarium* inoculation. The inoculation test was repeated at least three times with more than six different plants in each treatment.

For tomato, seeds of cv. Momotaro were germinated for six days, under the controlled environmental conditions mentioned above, then seedlings were transplanted to commercial garden soil for flowers and vegetables (Green Grow, Okayama, Japan). The inoculation with *R. irregularis* (100 spores/plant) or *G. margarita* (50 spores/plant) was conducted by mixing with soil. Plants were grown in the same controlled environmental conditions described above. Inoculation of the pathogen *Fusarium oxysporum* f. sp. *lycopersici* (FOL) strain JCM12575 race 2, the causal agent of *Fusarium* wilt, was performed as previously described (Egusa et al. 2019), with some modifications. Two-week-old tomato seedlings (after transplanting) were inoculated with 1 ml of a bud cell suspension (5×10^7^ cells/ml). Inoculated plants were maintained at 28°C for 14 h under light and at 18°C for 10 h in the dark. After 4 weeks, disease severity was assessed by counting the number of browning vascular bundles. The inoculation test was repeated at least 7 times with more than 4 independent plants in each treatment.

### Statistical analysis

Statistical analysis was performed using R software version 4.4.1 unless stated otherwise. The Welch’s *t*-test was used to compare two groups, and Tukey’s HSD test was used for multiple comparisons. For the *Fusarium* inoculation test in tomato, statistical significance in disease severity categorized by browning bundle was assessed using the Kruskal–Wallis test in RStudio (version 2022.07.2).

## Results

### Differences in shoot growth of *Rhizophagus-* and *Gigaspora*-colonized *Lotus japonicus* and tomato

**Figure 1:**
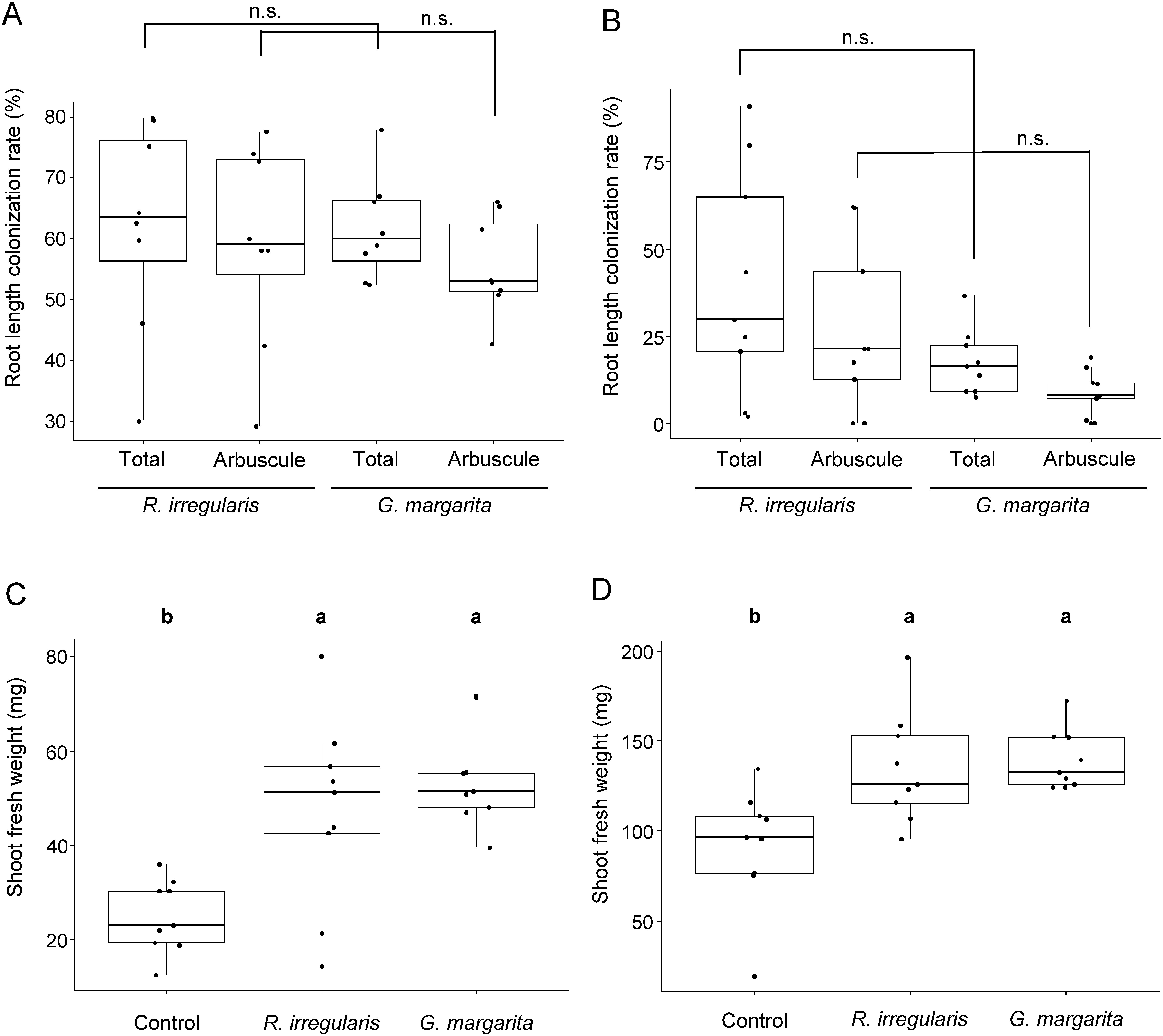
Figure 1. Fungal colonization rates and shoot growth in *Lotus japonicus* and tomato (*Solanum lycopersicum* L. cv. Micro-Tom) colonized by arbuscular mycorrhizal fungi. The root length colonization rates of *Rhizophagus irregularis* or *Gigaspora margarita* in *L. japonicus* (A) and tomato (B) at 6 weeks post-inoculation (wpi). Total, the percentage of all hyphal structures; Arbuscule, the percentage of arbuscules formed in cortical cells. There were no statistically significant differences (n.s.) in the colonization rates between *R. irregularis* and *G. margarita* (Welch’s *t*-test: *p*<0.05). Error bars show standard errors (A, *n*=8; B, *n*=9). Shoot fresh weight of *L. japonicus* and tomato colonized by *R. irregularis* or *G. margarita* in *L. japonicus* (C) and tomato (D) at six wpi. Control, non-colonized roots; *R. irregularis*, *R. irregularis*-colonized roots; *G. margarita*, *G. margarita*-colonized roots. Different lower-case letters indicate statistically significant differences (Tukey’s HSD: *p*<0.05). Error bars show standard errors (*n*=9).

AM morphotypes are basically determined by the phylogeny of the host plant (Smith and Smith 1997). The leguminous model plant *L. japonicus* exhibits an *Arum*-type AM by colonization of phylogenetically distant Glomeromycota fungi, such as *R. irregularis* and *G. margarita* (Supplementary Figure S1) (Tominaga et al. 2021; Venice et al. 2021). As an exception, tomato can form AMs with different morphotypes, such as the *Arum*-type AM formed by *R. irregularis* and *Paris*-type AM formed by *G. margarita* (Tominaga et al. 2022). Using these plant-AMF combinations, effects of the inoculated AMF identity and the resulting AM morphotype on shoot growth under phosphate starvation were examined. There were no significant differences in the colonization rates of *Rhizophagus*- and *Gigaspora*-colonized roots between *L. japonicus* and tomato (Figure 1A, B). AMF root colonization significantly promoted shoot growth in both *L. japonicus* and tomato, independent of the AMF identity (Figure 1C, D). The results obtained for tomato were similar to those obtained in our previous study (Tominaga et al. 2022). However, significant differences in shoot growth between *Rhizophagus*- and *Gigaspora*-colonized *L. japonicus* were observed after multiple independent experiments were conducted (Supplementary Figure S2).

**Figure 2:**
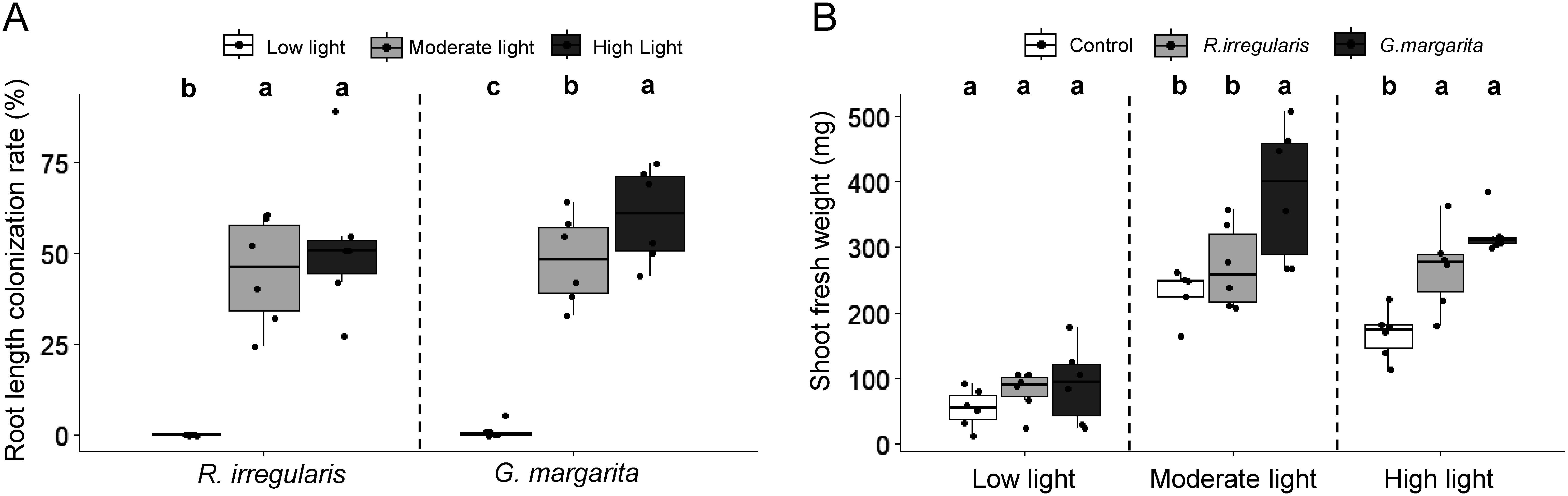
Figure 2. Effects of different light intensities on shoot growth and colonization rates in tomato (*Solanum lycopersicum* L. cv. Sugar Lump) inoculated with arbuscular mycorrhizal fungi (AMF). The root length colonization rates (A) and shoot fresh weight (B) of tomato plants, whose roots were inoculated with *Rhizophagus irregularis* or *Gigaspora margarita*, were grown under three light intensities (high: 330 µmol m^−2^ s^−1^, moderate: 80 µmol m^−2^ s^−1^, low: 5 µmol m^−2^ s^−1^) 6 weeks after AMF inoculation. Control, non-colonized roots; *R. irregularis*, *R. irregularis*-colonized roots; *G. margarita*, *G. margarita*-colonized roots. Different lower-case letters indicate statistically significant differences (Tukey’s HSD: *p*<0.05). Error bars show standard errors (*n*=6).

Micro-Tom, a dwarf cultivar of tomato (Martí et al. 2006), was used for methodological consistency with our previous study, which also used this tomato cultivar (Tominaga et al. 2022). There was no significant difference in shoot growth in Micro-Tom tomato plants colonized by *G. margarita* or *Rhizophagus* (Figure 1D). To assess whether this was a result specific to this cultivar, the same experiments were conducted with another cultivar, Sugar Lump, which is a German heirloom cherry tomato variety. In addition, the effect of light intensity, which has been shown to impact the colonization rate of AMF and promote shoot growth in response to AMF colonization (Wang et al. 2023), was tested. Among three different light conditions, roots were effectively colonized by both AMFs under moderate and high light intensities, but not under the weak light intensity (Figure 2A). Root colonization by *G. margarita* under the high light intensity was significantly higher than under the moderate light intensity (Figure 2A). Similarly, shoot growth of *Gigaspora*-colonized tomato plants was significantly higher than that of *Rhizophagus*-colonized plants under the conditions where AMF successfully colonized tomato roots (Figure 2B). These results indicate that the level of growth promotion caused by root colonization of both AMFs under a state of phosphate starvation was different depending on the colonized AMF identity, and independent of the resulting AM morphotype that formed.

### Comparative transcriptome analysis of *Rhizophagus*- and *Gigaspora*-colonized *Lotus japonicus* and tomato roots

To elucidate the molecular mechanism underlying the differences in growth in response to the colonized AMF identity, RNA extracted from *L. japonicus* roots colonized by *R. irregularis* and *G. margarita* were subjected to RNA-sequencing, corresponding to our previous analysis of tomato (Tominaga et al. 2022). Compared with control roots, 231 DEGs were identified in *Rhizophagus*-colonized roots, including 174 upregulated and 57 downregulated DEGs (Supplementary Table S2). In the case of *Gigaspora*-colonized roots, 8194 DEGs, including 4135 upregulated and 4059 downregulated DEGs, were identified (Supplementary Table S3). Among all DEGs of *Rhizophagus*-colonized roots, over 80% ovrelapped with those of *Gigaspora*-colonized roots (Supplementary Figure S3). In addition, *Gigaspora*-colonized roots exhibited a greater number of specific DEGs than *Rhizophagus*-colonized plant roots.

**Figure 3:**
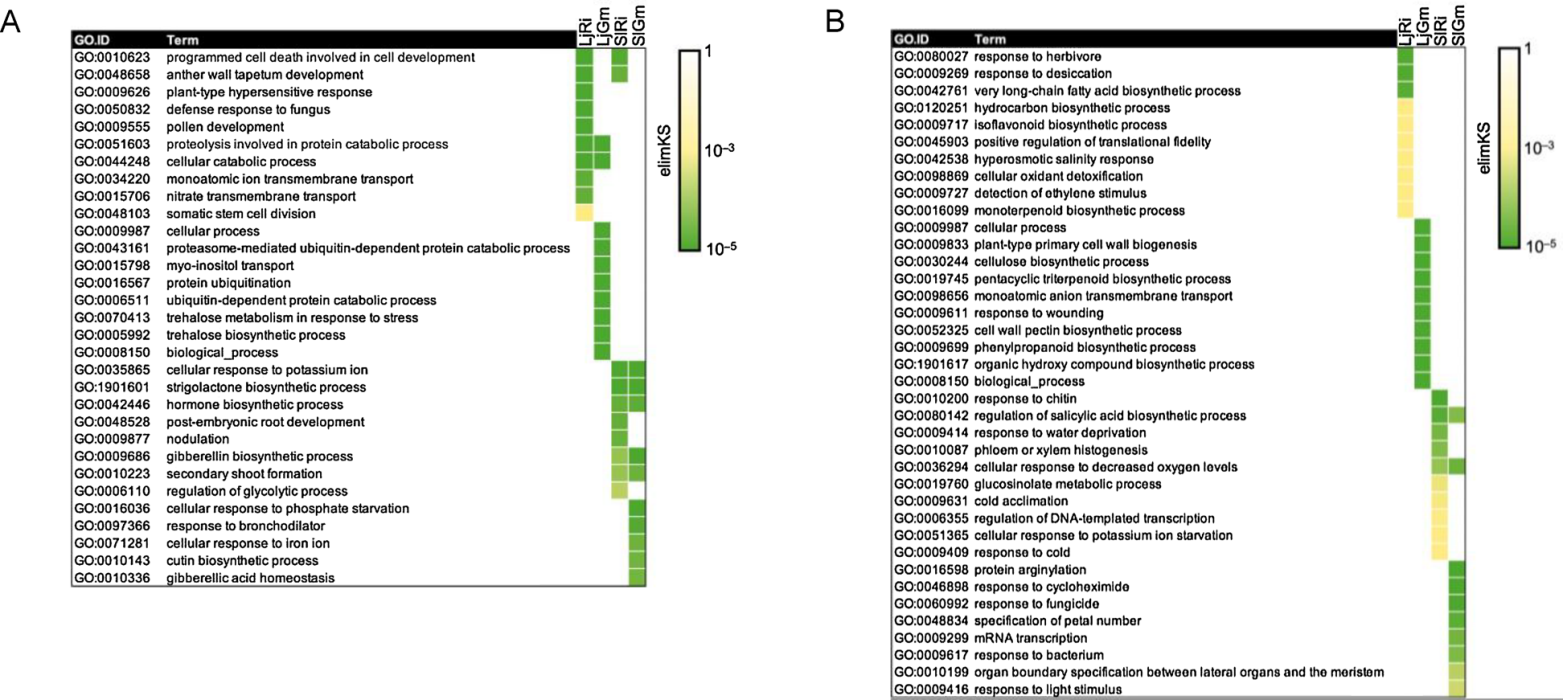
Figure 3. Gene ontology (GO) enrichment analysis of differentially expressed genes (DEGs) in *Lotus japonicus* and tomato roots colonized by arbuscular mycorrhizal fungi (AMF). Enriched GO terms related to biological process for the upregulated (A) and downregulated (B) DEGs after root colonization by *Rhizophagus irregularis* or *Gigaspora margarita* were identified with a cut-off of elim-Kolmogorov–Smirnov (KS) value <0.01. Top 10 GO terms based on elimKS values are shown for each plant-AMF combination. LjRi: DEGs in *R. irregularis*-colonized *L. japonicus*; LjGm: DEGs in *G. margarita*-colonized *L. japonicus*; SlRi: DEGs in *R. irregularis*-colonized tomato; SlGm: DEGs in *G. margarita*-colonized tomato. The complete lists of enriched GO terms are found in Supplementary Tables S4–7.

GO enrichment analysis was then conducted for DEGs identified in AMF-colonized *L. japonicus* roots (Supplementary Tables S2, S3) and tomato roots (Tominaga et al. 2022). In *L. japonicus* roots colonized by *R. irregularis*, 33 overrepresented GO terms were identified, 20 and 13 of which corresponded to upregulated and downregulated genes, respectively (Supplementary Table S4). In *Gigaspora*-colonized *L. japonicus* roots, 171 and 163 GO terms were identified in upregulated and downregulated genes, respectively (Supplementary Table S5). Only two terms (protein catabolic process) of the top 10 terms based on the elimKS value were shared between *Rhizophagus*- and *Gigaspora*-colonized *L. japonicus* roots (Figure 3A). In tomato roots, 36 and 25 GO terms were identified in *R. irregularis*-colonized roots, and 45 and 24 terms were identified in *G. margarita*-colonized roots among upregulated and downregulated DEGs, respectively (Supplementary Tables S6, S7). Among the GO terms identified in tomato, seven overrepresented terms were in common between the two types of AMF colonization (Figure 3A, B), including GO terms associated with plant hormone biosynthesis (e.g., “strigolactone biosynthesis process (GO: 1901601)”, “gibberellin (GA) biosynthesis process (GO: 0009686)”) among the upregulated DEGs (Figure 3A), and the defense-related GO term “regulation of salicylic acid (SA) biosynthetic process (GO: 0080142)” among the downregulated DEGs (Figure 3B).

In contrast, none of the top 10 GO terms was in common among all four host plant-AMF combinations (Figure 3). GO terms involved in development (GO: 0010623, GO: 0048658, GO: 0009555, GO: 0048528, and GO: 0010223), trehalose biosynthesis/metabolism (GO: 0070413, GO: 0005992), and phosphate starvation (GO: 0016036) were overrepresented in upregulated DEGs of either AMF-colonized plant roots (Figure 3A). A GO term for the defense response to fungus (GO: 0050832) was also enriched among upregulated DEGs in *Rhizophagus*-colonized *L. japonicus* roots (Figure 3A). Among the downregulated DEGs, GO terms associated with disease resistance (GO: 0080027, GO: 0009717, GO: 0009699, GO: 0010200, GO: 0019760, GO: 0060992, GO: 0009617) were widely overrepresented (Figure 3B). In addition, GO terms associated with cell wall function (GO: 0009833, GO: 0030244, GO: 0052325), which were reported to associate with defense response (Bacete et al. 2018; Maherali and Klironomos 2007; Molina et al. 2021), were found in *Gigaaspora*-colonized *L. japonicus* roots (Figure 3B).

### Differences in protective effects by AMF root colonization against *Fusarium* diseases

**Figure 4:**
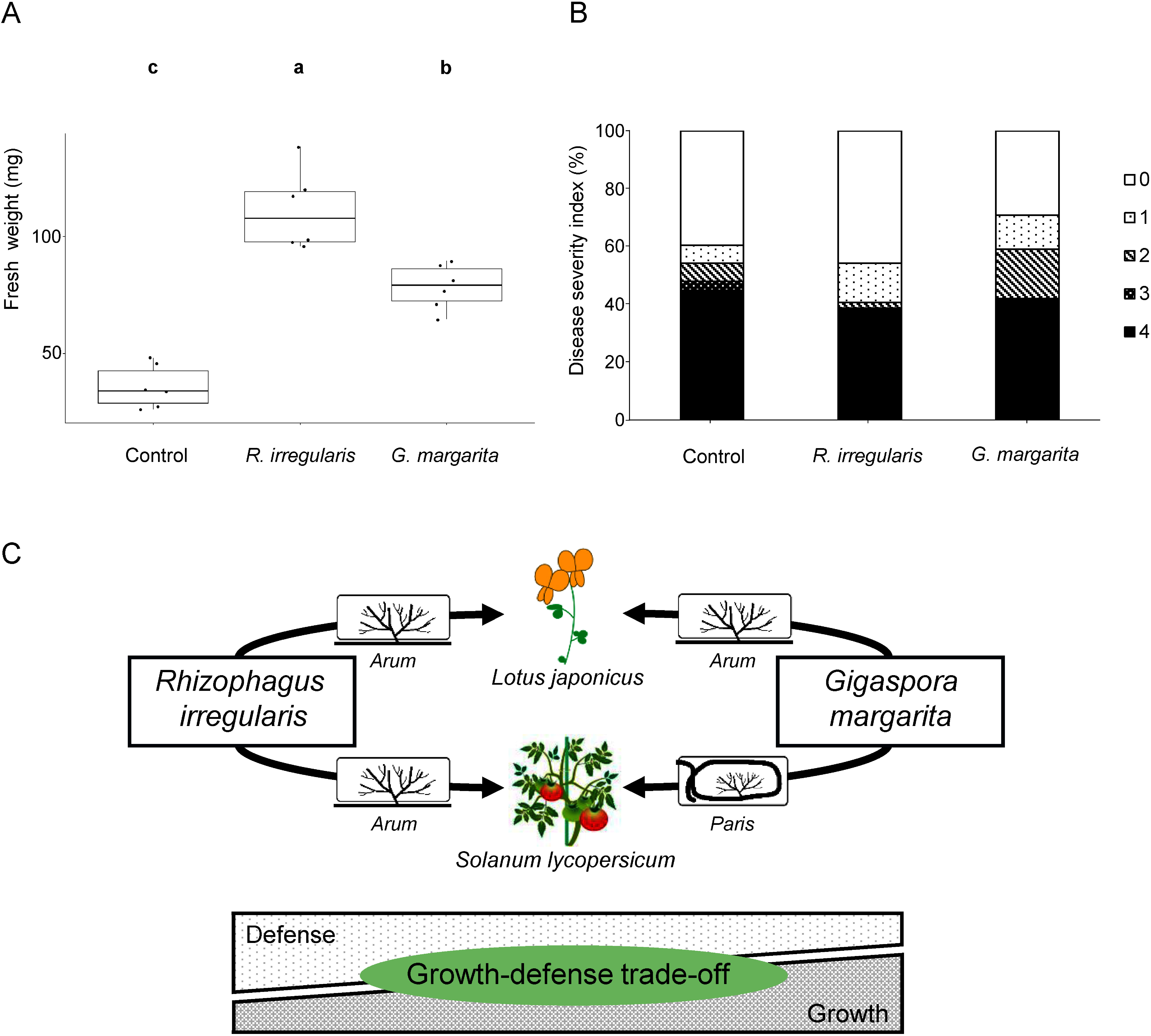
Figure 4. Effects of root colonization by arbuscular mycorrhizal fungi (AMF) on resistance against *Fusarium* diseases in *Lotus japonicus* and tomato. (A) The severity of *Fusarium* root rot disease in *L. japonicus*. *Lotus japonicus* plants were cultured with AMF, *Rhizophagus irregularis* or *Gigaspora margarita*, for 6 weeks prior to *Fusarium* inoculation. Inoculation of *Fusarium solani-melongenae* was conducted by soil drenching, and shoot fresh weight was measured 3 weeks after *Fusarium* inoculation. Different lower-case letters indicate statistically significant differences (Tukey’s HSD: *p*<0.05). Error bars show standard errors (*n*=6). (B) The severity of *Fusarium* wilt disease in tomato. Tomato (*Solanum lycopersicum* L. cv. Momotaro) plants were cultivated with an AMF, *R. irregularis* or *G. margarita*, for 2 weeks prior to *Fusarium* inoculation. Tomato plants were then inoculated with *Fusarium oxysporum* f. sp. *lycopersici*, and symptoms were observed 4 weeks after *Fusarium* inoculation. Disease symptoms were characterized by the number of browning vascular bundles as follows: 0, no symptoms; 1, one browning vascular bundle; 2, two browning vascular bundles; 3, three browning vascular bundles; 4, four browning vascular bundles or plant death. At Kruskal–Wallis test, significant differences in disease symptoms among treatment were not found. (C) Schematic diagram of growth-defense tradeoffs in AMF-colonized plants. In *L. japonicus* and *S. lycopersicum*, an *Arum*-type AM is formed by the colonization of *R. irregularis*. In contrast, *G. margarita* forms *Arum*-type and *Paris*-type AMs in *L. japonicus* and *S. lycopersicum*, respectively. Regardless of AM morphotype, the level of growth-defense tradeoff is fine-tuned, depending on AMF identity.

Since GO terms associated with defense response were widely enriched in upregulated or downregulated DEGs identified in AMF-colonized *L. japonicus* and tomato roots, the increased disease resistance by AMF root colonization, which is a known as MIR (Cameron et al. 2013), was then examined in the same plant-AMF combinations as above, but using the causal agents of *Fusarium* diseases. In *L. japonicus*, FSO caused *Fusarium* root rot (Takeuchi et al. 2007). Disease severity was assessed based on shoot growth 3 weeks after FSO inoculation. The colonization of *L. japonicus* roots by *R. irregularis* and *G. margarita* suppressed the inhibition of shoot growth caused by FSO infection (Figure 4A). The protective effect of *Rhizophagus*-colonized roots was more effective than in *G. margarita*-colonized roots.

For tomato, the assay included FOL and a cultivar (Momotaro) susceptible to FOL. Although there was no statistically significant difference in disease symptoms among treatment, the infection rate of FOL in *Rhizophagus*-colonized plants was not different from control plants, but the disease symptoms tend to be more severe in control plants than in *Rhizophagus*-colonized plants (Figure 4B). Colonization of tomato roots by *G. margaria* made plants more susceptible to FOL infection, although the disease symptoms appeared to be more moderate than the control. These results indicate that root colonization by either AMF suppressed the progression of *Fusarium* diseases, but that the protective effect on *Fusarium* diseases by root colonization by *R. irregularis* was higher than by root colonization by *G. margarita*.

## Discussion

Plants invest as much as 20% of their carbon sources fixed during photosynthesis to maintain their mutual association with AMF (Jakobsen 1995). Thus, plants need to balance the allocation of their remaining limited resources during an AM symbiosis. Root colonization by AMF provides host plants with various beneficial traits, such as the promotion of growth and resistance to abiotic and biotic stresses (Shi et al. 2023). However, a beneficial change in one trait is often linked to detrimental changes in another traits (Stearns 1989). In this study, two agronomically beneficial traits, plant growth and induced disease resistance, caused by AM symbioses, were examined in *L. japonicus* and tomato inoculated with two phylogenetically distal AMF, *R. irregularis* and *G. margarita*. *Gigaspora*-colonized plants showed more growth promotion than *Rhizophagus*-colonized plants, whereas the levels of induced resistance against *Fusarium* diseases in *Rhizophagus*-colonized plants was higher than in *Gigaspora*-colonized plants.

The presence of AM morphotype-dependent specific responses in plants was previously indicated (Gao et al. 2004; Hong et al. 2012; Tominaga et al. 2022). In tomato, two defense-related genes, *PR-1* and *CHI3*, were transiently upregulated in a *Paris*-type AM, whereas the examined defense-related genes were not induced in an *Arum*-type AM (Gao et al. 2004). Upregulated transcriptional responses associated with defense and GA biosynthesis specifically occurred in a *Paris*-type AM, while colonization rate, shoot biomass, and upregulation of AM-related genes were similarly observed in both types of AMs formed in tomato (Tominaga et al. 2022). In this study, similar differences in the levels of shoot growth promotion and disease resistance induced by AMF colonization were observed, but this depended on the colonized AMF identity in *L. japonicus* and tomato, but was not associated with formed AM morphotype. However, the transcriptional changes in common to both plants inoculated with the same AMF were very limited, which is supported by findings in the past report (Sugimura and Saito 2017). A similar comparative transcriptome analysis was conducted in this study, which indicated that most DEGs induced by AMF inoculation were not in common, and no shared GO terms were found in the roots of both *R. irregularis*-colonized *L. japonicus* and tomato. Thus, transcriptional responses to AMF root colonization depend on the host plant species except for a certain proportion of genes that are essential for the AM symbiosis.

Generally, AM symbiosis enhances host plant growth, especially under phosphate starvation, although there are negative effects on plant growth because plants do not gain a net benefit only from that symbiosis (Jin et al. 2017). In this study, shoot growth was significantly promoted in all host plant-AMF combinations, although differences between *Rhizophagus*- and *Gigaspora*-colonized plants were subtle when *L. japonicus* and Micro-Tom tomato were used, whose shoot biomass is tiny. However, when light intensity and the tomato cultivar were exchanged, clear differences were revealed: a higher root colonization rate and shoot biomass in *Gigaspora*-colonized tomato than in *Rhizophagus*-colonized one. *Gigaspora margarita* belongs to the Gigasporaceae, which is an early diverging AMF group and is clearly separated from *R. irregularis*, which belongs to the Glomeraceae (Krüger et al. 2012). It has been claimed that AMF belonging to the Gigasporaceae exchange more nutrients with their host plants than those belonging to the Glomeraceae (Powell et al. 2009; Sikes et al. 2009). Thus, different abilities to allocate nutrients would contribute to differences in the levels of shoot growth observed between *Rhizophagus*- and *Gigaspora*-inoculated plants. The analysis suggested the involvement of plant hormones, strigolactone and GA, trehalose, and phosphate starvation in the transcriptional responses in AMF-colonized plants. These responses related to phytohormones and phosphate starvation are widely acknowledged to participate in AM symbiosis (Miura et al. 2024; Shi et al. 2023). Trehalose metabolism was proposed to be involved in AM symbiosis in *Daucus carota* and *Eustoma grandiflorum* (Tominaga et al. 2021). Therefore, the transcriptional reprogramming of genes involved in these responses may trigger the promotion of AM symbiosis, which would increase plant shoot growth.

The colonization of *L. japonicus* and tomato roots by both AMF induced significant resistance against two *Fusarium* diseases, FOL and FSO. This is consistent with a report in which AMF-colonized plants showed increased tolerance/resistance to diverse soil-borne microbial pathogens, including *Fusarium* (Fiorilli et al. 2024). However, in this study, *Rhizophagus*-colonized plants, *L. japonicus* and tomato, showed more resistance to *Fusarium* than *Gigaspora*-colonized plants. Similarly, in *Plantago lanceolata*, symbiosis with AMF in the Gigasporaceae reduced infection by *F. oxysporum* or *Pythium* sp. relative to AMF in the Glomeraceae (Maherali and Klironomos 2007). The differences in pathogen infection by these AMF families may be as a result of their distinct life-history strategies: AMF in Glomeraceae rapidly colonize plant roots with hyphae while AMF in Gigasporaceae slowly colonize the outside of roots with hyphae (Hart and Reader 2002). In contrast, the transcriptome analysis in this study revealed the downregulation of genes associated with disease resistance in AMF-colonized both plant roots. In addition, GO terms associated with cell wall function were overrepresented in downregulated DEGs in AMF-colonized plant roots. Cell wall biogenesis, composition, and integrity are involved in plant disease resistance (Bacete et al. 2018; Maherali and Klironomos 2007; Molina et al. 2021). Since AMF must overcome the plant immune system for their successful colonization (Shu et al. 2023), AMF would induce transcriptional reprogramming to suppress host defense responses by introducing effectors (He et al. 2020). In addition, the biosynthesis of salicylic acid (SA) seems to have been downregulated by AMF colonization in this study. Jasmonates are known to accumulate during AM symbioses and are involved in disease resistance against necrotrophic pathogens such as *Fusarium* (Cameron et al. 2013; Pieterse et al. 2012). Because many reports describe an antagonistic effect between SA- and JA-signaling pathways, the downregulation of SA biosynthesis may contribute to MIR triggered by AMF colonization.

In summary, we revealed that AMF identity, and not AM morphotype, is involved in the traits, shoot growth promotion and induced disease resistance against *Fusarium*, commonly induced by AMF colonization. Notably, shoot growth promotion was inversely related to induced disease resistance (Figure 4C). This could be explained by well-known growth-defense tradeoffs (Huot et al. 2014). During an AM symbiosis, the status of resource restriction is more severe. Therefore, AMF-colonized plants must fine-tune the balance of limited resources to optimize fitness, leading to differences in these two traits. There are tradeoffs between AM symbiosis and agronomic traits such as plant growth promotion or disease resistance have been found and argued so far (Jacott et al. 2017). However, tradeoffs between these agronomic traits were evidenced in this study. This study will shed light on understanding the mechanism underlying the importance of two agronomic traits, plant growth and induced plant disease resistance, when triggered by root colonization of phylogenetically distal AMF.

## Accession numbers

The raw read data obtained by RNA-sequencing were deposited in the DNA Data Bank of Japan under the BioProject accession number PRJDB18939.

## Description of supplementary files

Supplementary Figure S1. Arbuscular mycorrhizae (AM) formed in *Lotus japonicus* roots colonized by *Rhizophagus irregularis* or *Gigaspora margarita*.

Supplementary Figure S2. Shoot fresh weight of *Lotus japonicus* colonized by arbuscular mycorrhizal fungi.

Supplementary Figure S3. Transcriptome analysis of *Lotus japonicus* roots colonized by arbuscular mycorrhizal fungi.

Supplementary Table S1. Summary of RNA-sequencing.

Supplementary Table S2. List of differentially expressed genes in *Lotus japonicus* roots colonized by *Rhizophagus irregularis.*

Supplementary Table S3. List of differentially expressed genes in *Lotus japonicus* roots colonized by *Gigaspora margarita.*

Supplementary Table S4. List of gene ontology terms enriched in differentially expressed genes found in *Lotus japonicus* roots colonized by *Rhizophagus irregularis.*

Supplementary Table S5. List of gene ontology terms enriched in differentially expressed genes found in *Lotus japonicus* roots colonized by *Gigaspora margarita.*

Supplementary Table S6. List of gene ontology terms enriched in differentially expressed genes found in tomato roots colonized by *Rhizophagus irregularis.*

Supplementary Table S7. List of gene ontology terms enriched in differentially expressed genes found in tomato roots colonized by *Gigaspora margarita.*
