# Supplementary material for: Arbuscular mycorrhiza-induced growth promotion and disease resistance are fine-tuned by growth-defense tradeoffs in Lotus japonicus and tomato
Source: Plant Biotechnol (Tokyo). 2025 Sep 25;42(3):289–98. doi: 10.5511/plantbiotechnology.25.0220a (PMC12573571; doi:10.5511/plantbiotechnology.25.0220a)
Supplement: Supplementary Data [file plantbiotechnology-42-3-25.0220a_s001.pdf]

Figure S1

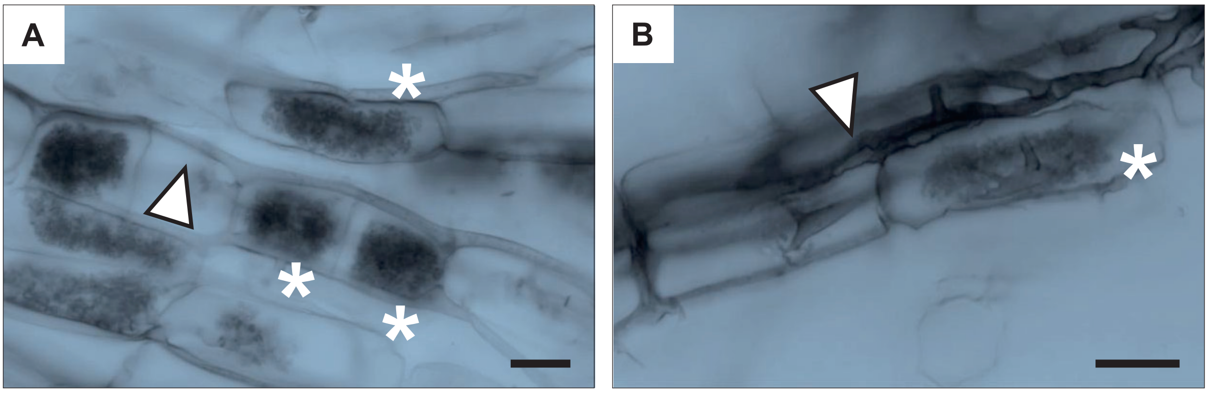

Supplementary Figure S1. Arbuscular mycorrhizae (AM) formed in *Lotus japonicus* roots colonized by *Rhizophagus irregularis* (A) or *Gigaspora margarita* (B). AM formed in *L. japonicus* roots were visualized by trypan blue staining. Scale bars, 20 µm. Arrowheads, intercellular hyphae; asterisks, arbuscules.

Figure S2

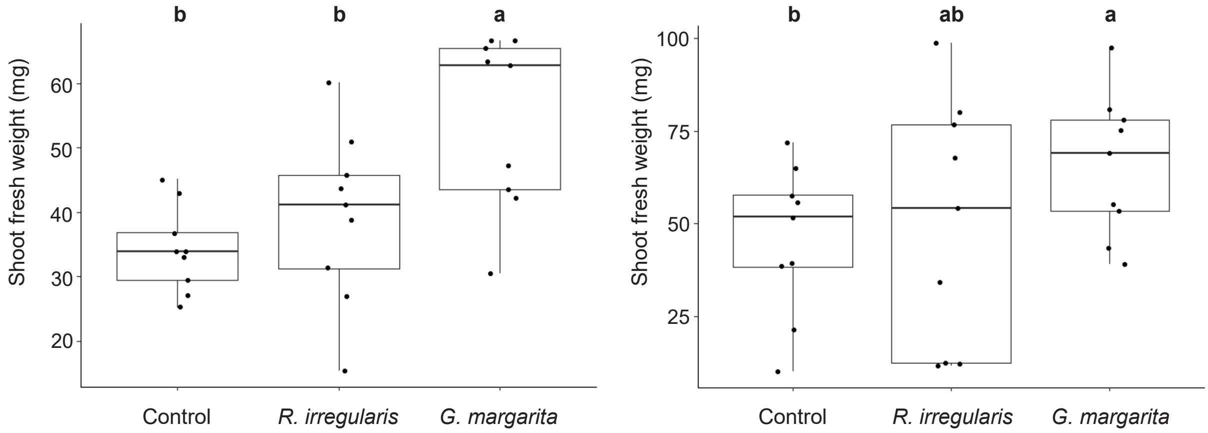

Supplementary Figure S2. Shoot fresh weight of *Lotus japonicus* colonized by arbuscular mycorrhizal fungi. The two representative results, which showed different tendencies from the result in Figure 1C, in seven independent trials are shown. All experimental procedures follow those described in Figure 1C. Control, non-colonized roots; *R. irregularis*, *Rhizophagus irregularis*-colonized roots; *G. margarita*, *Gigaspora margarita*-colonized roots. Different lower-case letters indicate statistically significant differences (Tukey's HSD: $p < 0.05$). Error bars show standard errors ($n = 9$).

Figure S3

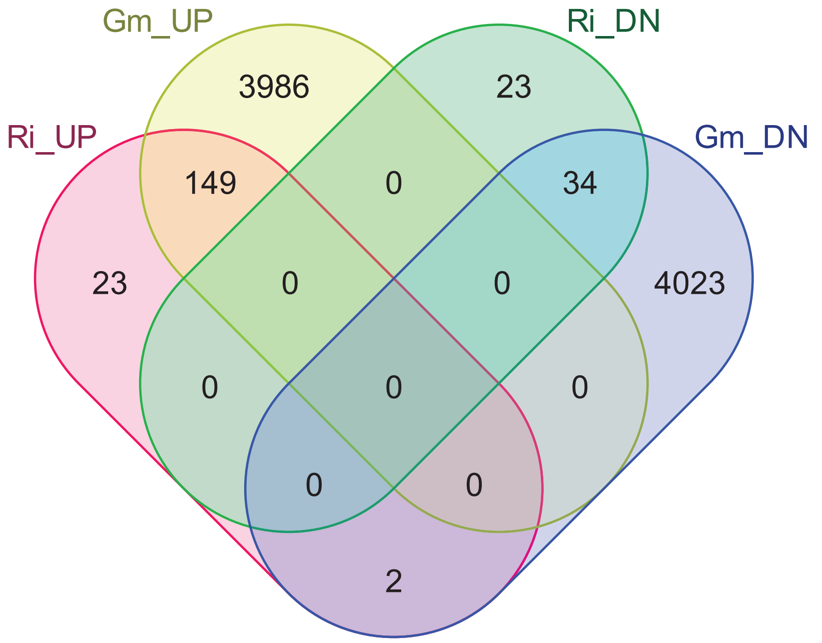

Supplementary Figure S3. Transcriptome analysis of *Lotus japonicus* roots colonized by arbuscular mycorrhizal fungi. Venn diagram of differentially expressed genes (DEGs) identified by a false discovery rate cutoff of < 0.05, shown in Table S2 and Table S3. Ri_UP, upregulated DEGs in *Rhizophagus irregularis*-colonized roots; Gm_UP, upregulated DEGs in *Gigaspora margarita*-colonized roots; Ri_DN: downregulated DEGs in *R. irregularis*-colonized roots; Gm_DN: downregulated DEGs in *G. margarita*-colonized roots.
